# DNA methylation changes during a sprint interval exercise performed under normobaric hypoxia or with blood flow restriction: A pilot study in men

**DOI:** 10.14814/phy2.16044

**Published:** 2024-06-07

**Authors:** Robert Solsona, Tom Normand‐Gravier, Fabio Borrani, Henri Bernardi, Anthony M. J. Sanchez

**Affiliations:** ^1^ Institute of Sport Sciences University of Lausanne Lausanne Switzerland; ^2^ Laboratoire Interdisciplinaire Performance Santé Environnement de Montagne (LIPSEM), UR 4640 University of Perpignan via Domitia, Faculty of Sports Sciences Font‐Romeu France; ^3^ DMEM, Université de Montpellier, INRAE Montpellier France

**Keywords:** altitude, epigenetics, occlusion, training

## Abstract

This crossover study evaluated DNA methylation changes in human salivary samples following single sprint interval training sessions performed in hypoxia, with blood flow restriction (BFR), or with gravity‐induced BFR. Global DNA methylation levels were evaluated with an enzyme‐linked immunosorbent assay. Methylation‐sensitive restriction enzymes were used to determine the percentage methylation in a part of the promoter of the gene‐inducible nitric oxide synthase (p‐iNOS), as well as an enhancer (e‐iNOS). Global methylation increased after exercise (*p* < 0.001; dz = 0.50). A tendency was observed for exercise × condition interaction (*p* = 0.070). Post hoc analyses revealed a significant increase in global methylation between pre‐ (7.2 ± 2.6%) and postexercise (10.7 ± 2.1%) with BFR (*p* = 0.025; *dz* = 0.69). Methylation of p‐iNOS was unchanged (*p* > 0.05). Conversely, the methylation of e‐iNOS increased from 0.6 ± 0.4% to 0.9 ± 0.8% after exercise (*p* = 0.025; *dz* = 0.41), independently of the condition (*p* > 0.05). Global methylation correlated with muscle oxygenation during exercise (*r* = 0.37, *p* = 0.042), while e‐iNOS methylation showed an opposite association (*r* = −0.60, *p* = 0.025). Furthermore, p‐iNOS methylation was linked to heart rate (*r* = 0.49, *p* = 0.028). Hence, a single sprint interval training increases global methylation in saliva, and adding BFR tends to increase it further. Lower muscle oxygenation is associated with augmented e‐iNOS methylation. Finally, increased cardiovascular strain results in increased p‐iNOS methylation.

## 1 INTRODUCTION

Epigenetics can be defined as changes in transcriptional activity that are not due to variations in the genetic sequence (Seaborne & Sharples, 2020). Among the epigenetic mechanisms, DNA methylation has been widely studied in recent years, and involves the attachment of a methyl group to cytosines within CpG dinucleotides, leading to the formation of 5‐methylcytosine. The regulation of gene expression through DNA methylation is transcendental for cellular function, both in physiological and pathological scenarios (Radak et al., 2023; Seaborne & Sharples, 2020; Seaborne, Strauss, Cocks, Shepherd, O'Brien, van Someren, et al., 2018; Widmann et al., 2019). Physical exercise transitory disrupts cellular homeostasis and imposes stress on the organism, prompting an adaptive response. This stress‐induced reaction is marked by the elevation of messenger RNA levels for numerous genes. DNA methylation plays a crucial role in regulating the cellular responses to exercise (Seaborne & Sharples, 2020; Solsona et al., 2022).

Since then, the effects of different exercise protocols on DNA methylation have been evaluated in skeletal muscle and white blood cells. Acute resistance exercise provokes changes in DNA methylation patterns in skeletal muscle biopsies, with over 17,000 CpG sites exhibiting differential methylation. The majority of these sites experienced a reduction in methylation (Seaborne, Strauss, Cocks, Shepherd, O'Brien, Someren, et al., 2018). Importantly, LINE‐1 (long interspersed nuclear element‐1) is a retrotransposon indicative of global methylation. Notably, Bagley et al. identified a reduction in LINE‐1 methylation levels within skeletal muscle biopsies following resistance exercise. This phenomenon was exclusively observed among resistance‐trained individuals, while global methylation remained unchanged in sedentary participants. This distinction implies different responses depending on the training status (Bagley et al., 2020). It is worth noting, however, that the methylation levels of genes associated to inflammation (i.e., interleukin‐6 [IL‐6] and tumor necrosis factor‐alpha [TNF‐α]) or hypertrophy (mechanistic target of rapamycin (mTOR) and Akt1) were unchanged in either group. Conversely, a recent study showed that acute resistance training involving eccentric constraints induced hypermethylation of IL‐6 and hypomethylation of TNF‐α in the skeletal muscle biopsies of untrained men (Hunter et al., 2023). Importantly, this study also highlighted a tissue‐specific response to exercise in terms of DNA methylation. For example, the baseline methylation levels of IL‐6 and TNF‐α in skeletal muscle and leukocytes were different, highlighting the tissue specificity of the epigenome.

A pioneering study delving into exercise‐induced DNA methylation alterations was published in 2012 by Barrès and colleagues. Their investigation unveiled that the promoters of certain metabolic genes undergo hypomethylation in an intensity‐depending manner during endurance exercise. This phenomenon was observed in skeletal muscle biopsies taken from healthy sedentary men and women, wherein several genes involved in aerobic function were upregulated when exercise was performed at high intensity, showcasing a potential link between exercise‐induced promoter hypomethylation and increased gene expression (Barrès et al., 2012). Sprint interval training (SIT) consists of the repetition of all‐out exercises (~30 s) interspersed with long recoveries (~4 min). This type of training induces skeletal muscle adaptations and gains in endurance performance in active men that are comparable to those observed with moderate‐intensity endurance training, but with considerably lower volume, resulting in enhanced time efficiency (Burgomaster et al., 2008; Gibala et al., 2006). In combination with blood flow restriction (BFR), this method allows trained cyclists to improve their V̇O_2_max (Mitchell et al., 2019; Taylor et al., 2016). Of note, the expression of hypoxia‐inducible factor‐1 alpha increased with BFR, suggesting that this method may cause a hypoxic stimulus to skeletal muscles (Taylor et al., 2016). An alternative BFR approach involves pedaling with the legs elevated above heart level to induce muscle ischemia. Referred to as gravity‐induced BFR (G‐BFR) by Preobrazenski and collaborators, this method was found to reduce muscle oxygenation during constant‐intensity submaximal cycling while eliciting a more pronounced increase in PGC1‐α messenger RNA (Preobrazenski et al., 2020). On the other hand, SIT in hypoxia has been shown to reduce arterial oxygen saturation without compromising power output, hinting at higher stress levels during the same exercise under hypoxic conditions (Solsona et al., 2021).

From a molecular point of view, inducible nitric oxide synthase (iNOS) is an enzyme that catalyzes the production of nitric oxide through L‐arginine oxidation. Polymorphonuclear cells can synthesize NO at rates similar to endothelial cells, thus suggesting a key role of iNOS in various physiological and pathological conditions (Sethi & Dikshit, 2000). It has been shown that infiltrating macrophages are responsible for iNOS expression in injured muscles (Rigamonti et al., 2013). The authors found that iNOS expression is required for effective regeneration of the tissue, as myogenic precursor cells in the injured muscle of iNOS^−/−^ mice fail to proliferate and differentiate. Importantly, NO is involved in the accumulation of free radicals after hypoxia reoxygenation in rat polymorphonuclear leukocytes, which suggests that iNOS may play an important role in the cellular response to hypoxia (Sethi et al., 1999). Regarding responses to exercise, the expression of iNOS was found to increase in the leukocytes of trained runners performing a half marathon (Niess et al., 2000). Furthermore, the addition of BFR to acute low‐intensity knee extensions was found to increase iNOS 24 h postexercise in skeletal muscle (Larkin et al., 2012). In untrained elders, 10 sets of 10 repetitions of eccentric leg‐press also upregulated iNOS in peripheral blood mononuclear cells (Jiménez‐Jiménez et al., 2008). The authors of this study reported an increase of both messenger RNA and protein levels of iNOS immediately and 3 h postexercise, suggesting that DNA methylation changes may play a role in iNOS expression. However, no study evaluated the impact of SIT protocols conducted in normobaric hypoxia or with BFR on DNA methylation. The impact of SIT on DNA methylation has been only studied after a biweekly training protocol of 3 months in sperm (Denham, O'Brien, Harvey, & Charchar, 2015) and after 1 month of training three times per week on leukocytes (Denham, O'Brien, Marques, & Charchar, 2015). The authors found decreased DNA methylation in several promoter regions, suggesting genome‐wide transcriptional activation. On the other hand, DNA methylation changes have been evaluated in salivary samples during a high‐altitude ascent (Childebayeva, Harman, et al., 2019). The results of this study show that the methylation levels of hypoxia‐sensitive genes change with short‐term altitude exposure, suggesting a role of epigenetics in altitude acclimatization. Furthermore, these changes were correlated with physiological parameters such as hemoglobin concentration and systolic blood pressure. The same research team evaluated DNA methylation in blood samples and found that Quechua individuals residing above 4000 m present higher methylation levels of LINE‐1 and lower methylation levels of EPAS1 compared to their lowlander counterparts (Childebayeva, Jones, et al., 2019). Therefore, epigenetic modifications, both at the global and gene‐specific levels, are associated with high‐altitude exposure. However, the acute changes in DNA methylation have not been studied after a single‐session SIT. Furthermore, the addition of different hypoxic stimuli could modulate exercise‐induced DNA methylation responses.

Saliva contains a mixture of two main cell types: approximately 70% of leukocytes and 30% of epithelial cells (Braun et al., 2019). Furthermore, a substantial proportion of CpG sites (88.5%) display strong correlations in the methylation patterns in saliva and blood samples (Smith et al., 2015). Similarly, other studies found similar DNA methylation patterns in whole blood and saliva (Liu et al., 2010; Thompson et al., 2013). Therefore, saliva was selected as the biofluid for conducting epigenomic analyses in this study.

Hence, the objective of this study was to examine DNA methylation changes in saliva after a session of SIT performed under systemic hypoxia or with BFR (HYP, BFR, and G‐BFR, respectively). Specifically, DNA methylation was analyzed globally, as well as in a segment of the iNOS promoter and an iNOS enhancer.

## 2 METHODS

### 2.1 Participants

Eleven moderately trained men (Table 1) performed the same SIT exercise under the four conditions: normoxia, normobaric hypoxia, BFR, and G‐BFR. They were healthy, did not reside in altitude or followed a prolonged sojourn in the last 6 months prior to the study. To avoid interactions with the current protocol, the participants did not consume dietary supplements, medication, or alcohol during the studied periods and 1 month before the beginning of this study. A standardized diet with 55% carbohydrate, 15% protein, and 30% fat was proposed to the participants for the day preceding the experiments. After being informed, they gave written consent to participate in this study, which was validated by the cantonal research ethics committee (“Commission cantonale d'éthique de la recherche sur l'être humain,” canton de Vaud, CER‐VD 2021–00597).

**TABLE 1:** Participant characteristics.

Body fat (%)	Weight (kg)	Height (cm)	Age (years)	Training volume (h/week)	Training frequency (sessions/week)
12.9 ± 1.8	74.6 ± 6.3	179 ± 6	25 ± 3	7.5 ± 3.8	4.1 ± 1.7

*Note*: Data are presented as mean ± standard deviation.

### 2.2 Study design

This study followed a randomized crossover design, with one session *per* week to avoid fatigue‐related bias. The normoxic condition (NOR) was performed below 400 m of altitude. For BFR, inflatable cuffs (SC12D, cuff size 13 × 85 cm) were placed around the most proximal part of the thighs and were connected to a rapid inflator system (E20/AG101 Rapid Cuff Inflation System, D.E Hokanson Inc., United States). Resting arterial occlusive pressure was measured before the trial with Doppler Ultrasound (EchoWave II 3.4.4, Telemed Medical Systems, Italy). Briefly, three measurements separated by 1 min allowed the determination of the cuff pressure that fully restricted blood flow in the femoral artery. Then, 60% of this pressure was applied immediately after the sprints for the first 2 min of recovery. This protocol was chosen based on two previous studies (Mitchell et al., 2019; Taylor et al., 2016). Hypoxia was performed in a normobaric hypoxic chamber (ATS altitude training, Australia). The fraction of inspired oxygen was 13% because, compared to lower stresses, additional benefits (such as increased Wingate test peak power) were found after a SIT protocol (Warnier et al., 2020). A structure was built for G‐BFR to allow participants to lay down and pedal horizontally. The position was adopted at the end of the warm‐up and kept during the whole session. Furthermore, participants were able to use vertical bars as handgrips and thus avoid body displacements. Sessions took place at the same time of the day to avoid the influence of the circadian cycle. Salivary samples (2 mL) were taken before the session, as well as 1 h after the end of the session. They were stored at −20°C for further analysis.

### 2.3 Exercise sessions

**FIGURE 1:**
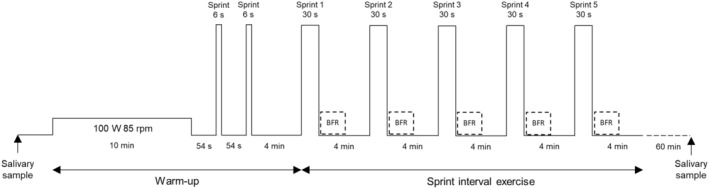
Representation of the exercise protocol. After a standardized warm‐up, a sprint interval training exercise consisting of five 30‐s sprints was performed under four conditions: BFR, blood flow restriction at 60% of resting arterial occlusive pressure during the first 2 min of recovery; G‐BFR, gravity‐induced blood flow restriction; hypoxia at FiO_2_ = 13%; and normoxia.

Exercise sessions took place in a laboratory environment, with an ergocycle (Lode Excalibur, Sport 911,905, Lode B.V., The Netherlands). After a 10‐min standardized warm‐up at 100 W, participants rested 54 s and performed a warm‐up sprint of 6 s two times. Then, 4 min of passive recovery preceded the first sprint out of five. Sprints were 30 s long and were separated by 4 min of passive recovery. The exercise protocol is represented in Figure 1. Participants were asked to maintain saddle contact and were verbally encouraged to provide maximal effort. A 3‐s countdown indicated the beginning of the sprints. Of note, the ergocycle was used in Wingate mode with a torque of 0.8 N/kg of body weight. Five‐minute recoveries were allowed between the sets. Peak power and mean power were recorded during each exercise session with the software of the cycling ergometer (Table 2).

**TABLE 2:** Mechanical, cardiorespiratory, and muscle oxygenation responses to various exercise conditions.

	BFR	G‐BFR	HYP	NOR
Peak power (W)	771 ± 114	705 ± 122^a^	783 ± 133	836 ± 176
Mean power (W)	561 ± 79	521 ± 84^b^	561 ± 69	583 ± 88
SpO_2_ (%)	98.7 ± 1.3	99.5 ± 0.5	89.7 ± 3.3^b^	98.3 ± 1.5
HR (bpm)	135 ± 15	122 ± 17^b^	138 ± 16	139 ± 16
HR_peak_ (bpm)	161 ± 10	156 ± 14^a^	163 ± 10	165 ± 8
TSI (%)	65 ± 7	53 ± 9^b^	60 ± 6	62 ± 6
VE (L)	335 ± 82	273 ± 77^b^	370 ± 86	326 ± 107
V̇E_peak_ (L·min^−1^)	149 ± 29	120 ± 24^b^	161 ± 27	156 ± 29
VO_2_ (L)	6.7 ± 1.1	6.4 ± 1.1^b^	7.0 ± 0.9	7.1 ± 0.9
V̇O_2peak_ (L·min^−1^)	3.85 ± 0.71	3.88 ± 0.69	3.41 ± 0.43^b^	4.00 ± 0.55
VCO_2_ (L)	7.1 ± 1.1^a^	7.0 ± 1.1^a^	7.2 ± 1.0	7.6 ± 1.1
V̇CO_2peak_ (L·min^−1^)	3.06 ± 0.49	3.06 ± 0.53	3.04 ± 0.44	3.20 ± 0.53

*Note*: Data are presented as mean ± standard deviation.

Abbreviations: BFR, blood flow restriction; G‐BFR, gravity‐induced blood flow restriction; HR, mean session heart rate; HR_peak_, peak heart rate; HYP, hypoxia; NOR, normoxia, SpO_2_, partial oxygen saturation; TSI, tissue saturation index during sprints; VCO_2_, average accumulated carbon dioxide exhaled; V̇CO_2peak_, peak carbon dioxide exhalation rate; VE, average accumulated ventilation during sprints and recoveries; V̇E_peak_, peak minute ventilation; VO_2_, average accumulated oxygen consumption during sprints and recoveries; V̇O_2peak_, peak oxygen consumption.

^a^ Significantly different from NOR (*p* < 0.05).

^b^ Significantly different from the other conditions (*p* < 0.05).

### 2.4 Cardiorespiratory responses assessment

Gas exchanges were evaluated breath‐by‐breath with a metabolic cart (Quark CPET, COSMED, Italy) that was calibrated before each test. Peak ventilation (V̇Epeak), peak oxygen uptake (V̇O_2_peak) and exhaled carbon dioxide (V̇CO_2_peak) were evaluated. The accumulated oxygen consumption was then multiplied by the caloric oxygen equivalent (21.131 kJ·L^−1^) to obtain aerobic energy consumption (Hebestreit & Beneke, 2007). Aerobic energy contribution was calculated as the ratio of aerobic energy consumption to total work, with an assumed mechanical efficiency of 23% (ÅStrand et al., 1960). A heart rate monitor (HRM3‐SS, Garmin, United Kingdom) was used to determine the heart rate during exercise and rest (HRex/rest), as well as peak heart rate (HRpeak). Muscle oxygenation was measured with an absolute near‐infrared spectroscopy probe (OxiplexTS, ISS, USA) that was placed on the distal part of the right vastus lateralis. A band was used to wrap the probe and avoid any displacement or light contamination.

### 2.5 DNA isolation and purification

An equal volume of lysis buffer was added to the salivary samples, as previously described (Quinque et al., 2006). The solution was incubated overnight at 53°C. The mixture was incubated for 10 min on ice after addition of 400 μL of 5 M NaCl. Next, the tubes were centrifuged for 10 min at maximal speed. Thereafter, the supernatant was transferred to new tubes and 800 μL of isopropanol were added. After 10 min at room temperature, they were centrifuged for 15 min at maximal speed. The supernatants were discarded, and the pellets were washed with 500 μL of 70% ethanol. Then, the pellets were dried and dissolved in 30 μL of distilled water. Thereafter, DNA was purified with columns from a commercial kit following the manufacturer's instructions (DNA Clean & Concentrator‐100, Zymo Research, USA, D4011). The concentration of DNA was measured by spectrophotometry (BioDrop DUO; BioDrop, Cambridge, United Kingdom).

### 2.6 Global DNA methylation levels

Quantification of global DNA methylation levels was performed with an enzyme‐linked immunosorbent assay (Kremer et al., 2012) (5‐mC DNA ELISA Kit, Zymo Research, USA, D5326). Hundred nanograms of DNA was denatured in a 100 μL 5‐mC coating buffer solution for 5 min at 98°C and immediately transferred on ice for 10 min. Then, the solution was incubated in the wells at 37°C for 1 h. Thereafter, three wash steps with 200 μL of 5‐mC ELISA Buffer preceded an incubation of 30 min at 37°C. Then, 100 μL of antibody mix (anti‐5‐methylcytosine: 1:2000; secondary antibody: 1:1000 both included in the Zymo kit D5326) was added to each well, and the plate was incubated at 37°C for 1 h. After three wash steps, 100 μL of HRP (included in the Zymo kit D5326) developer was added to each well, and absorbance was measured at 450 nm after 60 min of incubation at room temperature. Samples were run in duplicate and the two measurements were averaged.

The percentage methylation was calculated using the logarithmic equation of the standard curve that was constructed with the positive and negative controls as follows:

$$\%5\mathrm{mC}=e^{\frac{\text{absorbance}-y_{\text{intercept}}}{\text{slope}}}*8.$$

Of note, 8 is the estimated fold difference between *E. Coli* (serving as controls) and human CpG density. Global methylation was evaluated in 10 participants.

### 2.7 Methylation‐sensitive restriction enzymes qPCR

Primers of a region of the inducible nitric oxide synthase promoter (p‐iNOS, −1527 bp at 5′ side) (de Vera et al., 1996) and a sequence of an enhancer positioned in the promoter region (e‐iNOS, −6514 bp at 5′ side) (Guo et al., 2007) were designed with Primer‐BLAST (Ye et al., 2012). Primers are presented in Table 3. The amplicon size for p‐iNOS and e‐iNOS was 590 bp and 201 bp, respectively. Different annealing temperatures were tested for each primer pair and the best temperature was 65°C for p‐iNOS and 60°C for e‐iNOS. Also, primer efficacy was tested using a 10‐fold serial dilution and was 97.1% and 101.6% for p‐iNOS and e‐iNOS, respectively.

**TABLE 3:** Primers for qPCR.

	Forward	Reverse
p‐iNOS	AAGTGAGAGGATGGACAGGGATTA	TCACCTTGCAGCTGGCTGCACTGCC
e‐iNOS	GCCTTCCTTTGACAGCTGAG	GGTTTAAAGAGAGCCAGTTTAAGGT

*Note*: p‐iNOS: promoter of inducible nitric oxide synthase; e‐iNOS: enhancer of inducible nitric oxide synthase.

First, 500 ng of DNA were incubated for 1 h at 37°C in a 20 μL solution containing 0.5 μL of methylation‐sensitive restriction enzymes (HpaII (Biolabs, R0171S) for p‐iNOS and HpyCH4IV (Biolabs, R0619S) for e‐iNOS), reaction buffer and nuclease‐free water. Following digestion, enzymes were immediately inactivated with a 20‐min incubation at 80°C and 65°C for HpaII and HpyCH4IV, respectively. A real‐time PCR quantification was then performed using sensiFast SYBR Hi‐Rox kit (Bioline, BIO‐92020) on an Applied Biosystems StepOnePlus Thermal cycling block. The qPCR was performed with 20 ng of DNA as follows: 2 min at 95°C, 40 amplification cycles at 95°C for 10 s, 60/65°C for 20 s, and 72°C for 30 s. ΔCt was the difference in Ct between the digested DNA and the undigested DNA. Percent methylation was calculated as follows:

$$\%\text{methylation}=100*2^{-∆\mathrm{Ct}}.$$

DNA methylation was evaluated in seven participants for e‐iNOS and eight participants for p‐iNOS.

### 2.8 Statistical analysis

Statistics were performed using Jamovi (version 1.6.23) and RStudio (version 2023.3.1.446). Outliers were removed prior to analysis. Linear mixed models were used to analyze the main effect of exercise, condition and the exercise × condition interaction. The cluster variable was the participant number, which was the random coefficient together with the intercept. Post hoc analysis was performed with Holm's correction to adjust *p*‐values. Cohen's *dz* was employed to denote effect sizes: trivial effect *d* < 0.10, small effect 0.10 ≤ *d* < 0.50, medium effect 0.50 ≤ *d* < 0.80, and large effect *d* ≥ 0.80 (Cohen, 1992, 2016). The choice of Cohen's dz was made because of the crossover design of the study, resulting in correlated samples (Lakens, 2013). All data are presented as mean and standard deviation. The difference in methylation between the pretraining and post‐training values was used to perform correlation tests with mechanical and physiological parameters. The normality of the variables was verified with the Shapiro–Wilk test. When variables were normally distributed, Pearson tests were used, otherwise Spearman tests were used. The significance was declared at the threshold of *p* < 0.05.

## 3 RESULTS

### 3.1 Global methylation

**FIGURE 2:**
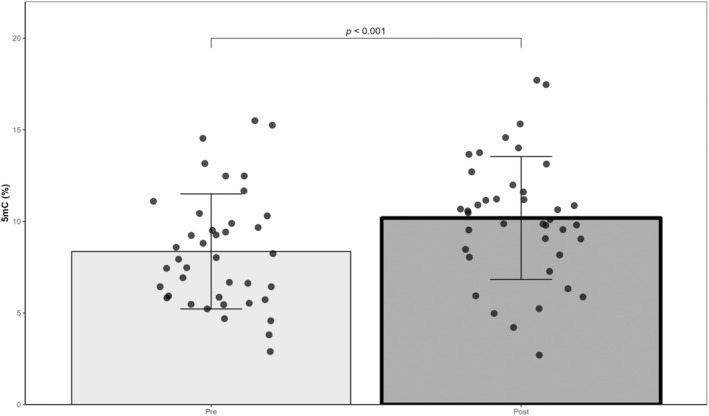
Changes in global DNA methylation level in response to exercise (*N* = 10, Pre: 40 samples, Post: 40 samples). 5mC: 5‐methylcytosine. Main effect of exercise was found (linear mixed model).

**FIGURE 3:**
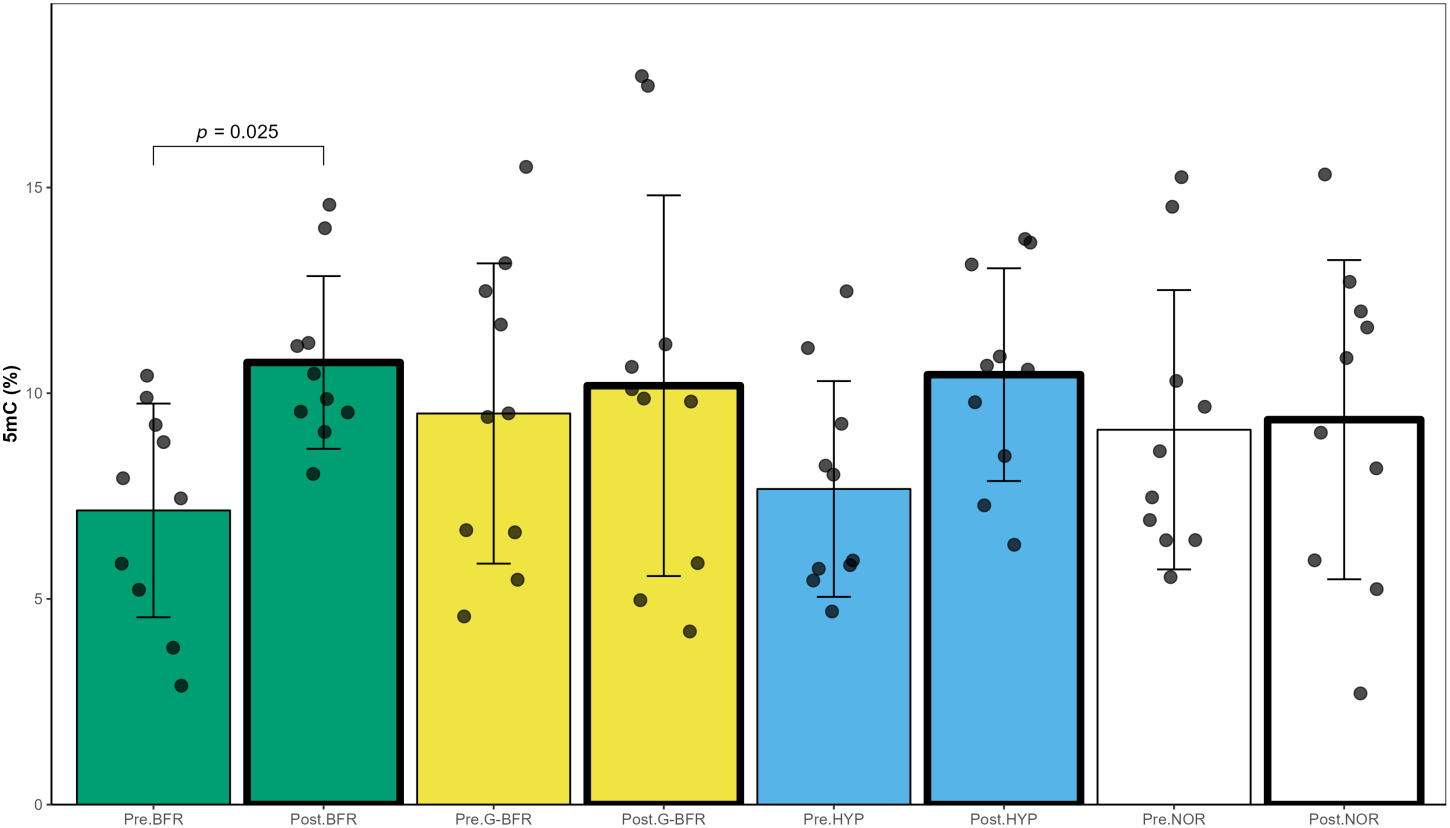
Changes in total DNA methylation level in response to exercise in normoxia (NOR), in hypoxia (HYP), with blood flow restriction (BFR), and with gravity‐induced blood flow restriction (G‐BFR) (*N* = 10, 10 samples per condition). Interaction: exercise × condition was found (linear mixed model).

The correlation coefficient between the logarithmic equation and the standard curve generated with the controls was 0.98 ± 0.01. According to linear mixed models, no effect of condition was found for global methylation (*p* = 0.617), but an effect of exercise was observed (*p* < 0.001; *dz* = 0.50). Global methylation increased from 8.4 ± 3.1% before exercise to 10.2 ± 3.4% 1 h after SIT (Figure 2). Furthermore, a trend was found for the exercise × condition interaction (*p* = 0.070). Post hoc analysis showed that BFR was responsible for this trend (Figure 3). Global methylation was higher post‐BFR compared to pre‐BFR (*p* = 0.025; *dz* = 0.69). Values were 7.2 ± 2.6% and 10.7 ± 2.1% for pre‐BFR and post‐BFR, respectively.

### 3.2 iNOS promoter and enhancer methylation

**FIGURE 4:**
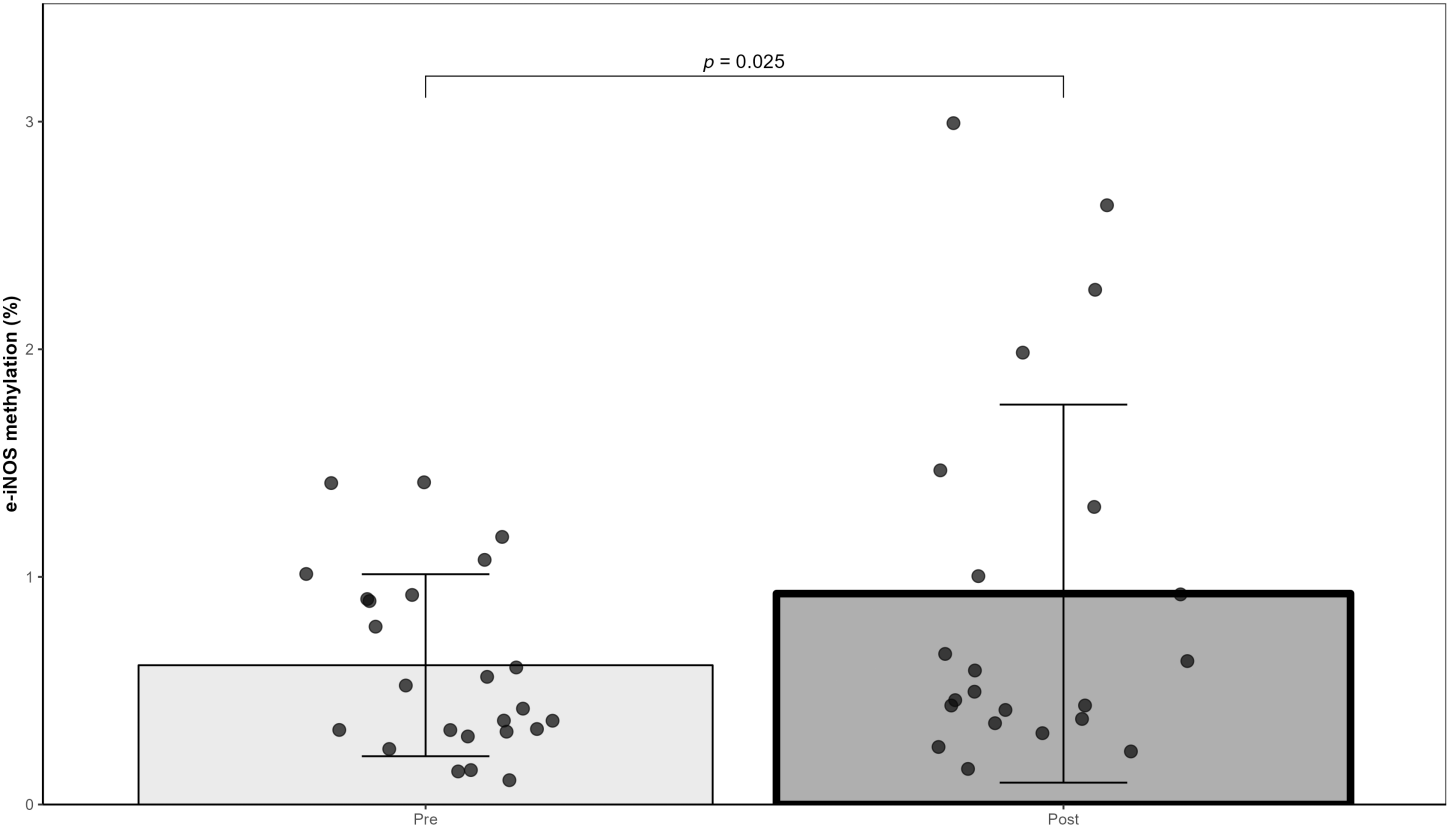
Changes in e‐iNOS methylation level in response to exercise (*N* = 7, Pre: 24 samples, Post: 22 samples). Main effect of exercise was found (linear mixed model).

The methylation of p‐iNOS did not present main effects of condition (*p* = 0.194), exercise (*p* = 0.231), or exercise × condition interaction (*p* = 0.163). Similarly, the methylation of e‐iNOS did not show a main effect of the condition (*p* = 0.313) or exercise × condition interaction (*p* = 0.746). However, the methylation level of e‐iNOS significantly increased after exercise (*p* = 0.025; *dz* = 0.41). Values were 0.6 ± 0.4% and 0.9 ± 0.8% for pre‐ and postexercise, respectively (Figure 4). Regarding p‐iNOS values were 9.7 ± 7.0% and 7.8 ± 5.8% before and after exercise, respectively. Percent methylation of p‐iNOS and e‐iNOS before and after exercise is presented in Table 4.

**TABLE 4:** p‐iNOS and e‐iNOS percent methylation in the different conditions pre‐ and postexercise.

	BFR		G‐BFR		HYP		NOR	
	Pre	Post	Pre	Post	Pre	Post	Pre	Post
p‐iNOS methylation (%)	11.1 ± 8.8	4.6 ± 2.2	6.1 ± 4.4	7.4 ± 7.5	8.8 ± 4.8	10.7 ± 6.1	12.2 ± 8.3	9.1 ± 5.6
e‐iNOS methylation (%)	0.6 ± 0.3	0.7 ± 0.8	0.5 ± 0.5	0.4 ± 0.2	0.6 ± 0.5	1.2 ± 0.9	0.7 ± 0.4	1.0 ± 1.0

*Note*: Data are presented as mean ± standard deviation.

Abbreviations: BFR, blood flow restriction; e‐iNOS, enhancer of the gene inducible nitric oxide synthase; G‐BFR, gravity‐induced blood flow restriction; HYP, hypoxia; NOR, normoxia; p‐iNOS, promoter of the gene inducible nitric oxide synthase.

### 3.3 Associations between DNA methylation and physiological data

**FIGURE 5:**
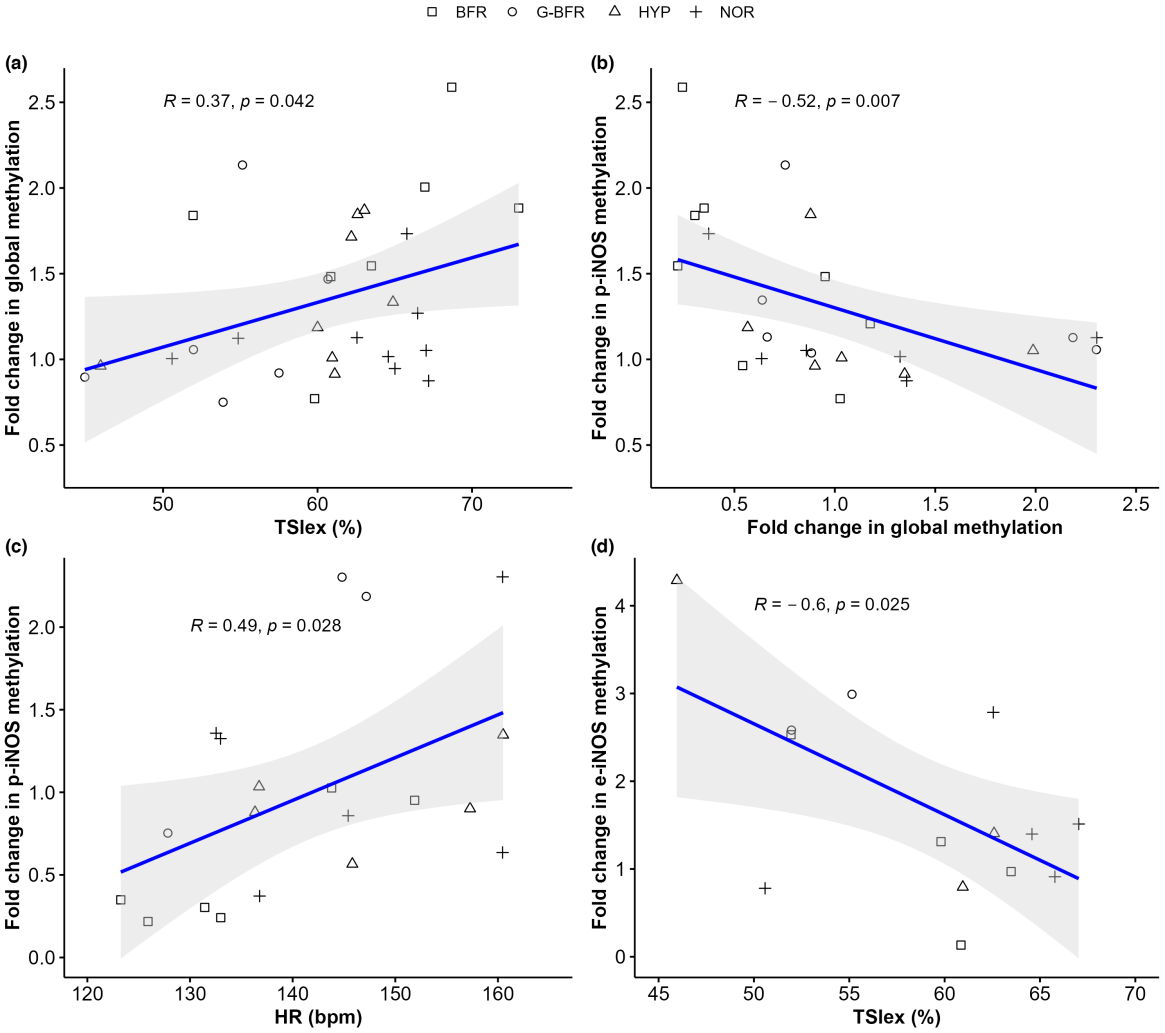
Significant correlations between methylation fold changes and physiological responses to sprint interval training. (a) Spearman correlation between fold change in global methylation and tissue saturation index during exercise (TSIex). Number of comparisons: 30. (b) Spearman correlation between fold changes in global methylation and fold changes in p‐iNOS methylation. Number of comparisons: 26. (c) Spearman correlation between fold changes in p‐iNOS methylation and average heart rate (HR). Number of comparisons: 20. (d) Pearson correlation between fold changes in e‐iNOS methylation and TSIex. Number of comparisons: 14. BFR, blood flow restriction; G‐BFR, gravity‐induced blood flow restriction; HYP, hypoxia, and NOR, normoxia.

Global methylation fold change was associated with mean tissue saturation index during exercise (*r* = 0.37; *p* = 0.042, Figure 5a). Global methylation fold change was also negatively correlated with p‐iNOS methylation fold change (*r* = −0.52, *p* = 0.007, Figure 5b). On the other hand, p‐iNOS methylation fold change was linked to mean session heart rate (*r* = 0.49, *p* = 0.028, Figure 5c). Finally, e‐iNOS methylation fold change was negatively correlated to mean tissue saturation index during exercise (*r* = −0.60, *p* = 0.025, Figure 5d).

## 4 DISCUSSION

This crossover study investigated the modifications in DNA methylation in response to an SIT exercise performed under systemic hypoxia or with BFR. Specifically, DNA methylation changes were measured both at the global level and within short sequences located in the iNOS promoter, as well as within a segment of an iNOS enhancer. The results showed that global methylation increased after exercise, and tended to increase more in BFR. After the exercise session, the methylation of p‐iNOS was unchanged, but the methylation level of e‐iNOS increased. However, no additional effect of the condition was found. Interestingly, both global and e‐iNOS methylation increased after exercise. However, the global DNA methylation changes were positively correlated to mean tissue saturation index during exercise, whereas the methylation changes of e‐iNOS displayed a negative association. Furthermore, the methylation fold change of p‐iNOS was correlated to mean session heart rate. Therefore, higher levels of muscle deoxygenation result in hypermethylation of e‐iNOS. Similarly, higher cardiovascular stress was associated with higher postexercise methylation values for p‐iNOS. Of note, even if the methylation of p‐iNOS did not significantly change after the session, fold changes in p‐iNOS methylation were negatively associated with global methylation levels. Therefore, although the conclusions regarding the results of correlations should be taken with caution, global methylation and the methylation of e‐iNOS are two candidates that respond oppositely to exercise‐induced deoxygenation. Lastly, the hypermethylation of p‐iNOS after exercise could be a marker of the cardiovascular strain.

Recently, Childebayeva and coworkers measured changes in total DNA methylation and the methylation level of genes involved in altitude acclimatization in saliva during an incremental ascent to high altitude (Childebayeva, Harman, et al., 2019). To the best of our knowledge, our study is the first to evaluate the effects of an acute session of exercise on total DNA methylation of human saliva. Regarding global methylation, the ELISA array analysis revealed that global methylation increased 1 h after the exercise. The ELISA method offers the advantage of identifying methylated cytosines while excluding those in the initial hypomethylation stage, unlike bisulfite‐based methods. Hence, it is an accurate method for global DNA methylation analysis (Kremer et al., 2012). In blood samples, a study compared the effects of two different workload‐matched cycling sessions (38 min at 50% of peak aerobic power or 6 × 3 min at 85% of peak aerobic power with 3 min of active recovery at 25% of peak aerobic power) on global DNA methylation (De Lazzari et al., 2021). In agreement with our results, global DNA methylation augmented 1 h after exercise. Of note, this study also used an ELISA array for the assessment of global DNA methylation. Strikingly, this increase of DNA methylation was only found in the low‐intensity trial and suggests that at submaximal intensities, continuous exercise may be more effective than intermittent exercise in inducing changes in global DNA methylation. Conversely, another study on blood samples evaluated DNA methylation changes with the 27k methylation array from Illumina. Global DNA methylation was unchanged in trained runners after a 120‐min run at 60% of V̇O_2_max followed by a 5 km time trial (Robson‐Ansley et al., 2014). In this study, samples were taken immediately and 24 h postexercise. Of note, the participants performed the exercise in fasting conditions. Remarkably, although the fold changes were small, the modification of the methylation status of 11 CpG sites in different genes was correlated with the levels of circulating IL‐6. Comparatively, the methylation of 662 regions was modified after a 15‐min cycling protocol at 80% of V̇O_2_max in the subcutaneous white adipose tissue of sedentary men (Fabre et al., 2018). Most DNA methylation changes happened within 20 min after the training session, rather than 60 or 240 min. This means that DNA methylation changes can be a very dynamic process. Many of these differentially methylated regions were found in gene promoters, due to the high CpG content they present. Interestingly, gene ontology enrichment analysis showed that the differentially expressed genes were related to inflammatory responses and the positive regulation of nitric oxide biosynthesis. Similar to our results, this observation suggests a role of iNOS methylation in modulating responses to exercise, but further research is necessary to elucidate the specific implications of these methylation changes in e‐iNOS. The study by Fabre and colleagues found no correlation between DNA methylation remodeling and gene expression (evaluated through RNA sequencing) (Fabre et al., 2018). The authors stated that this discrepancy could be attributed to the variable time course of gene expression, which was only quantified 4 h postexercise. On the other hand, the association between CpG methylation changes and gene expression may depend on the genomic region where DNA changes occur. Furthermore, this relationship is not always evident and the involvement of chromatin remodeling seems a better predictor of gene expression (Wagner et al., 2014). Taken together, these data strongly suggest the timing and the modality of the exercise appear as critical factors that influence the modulation of total DNA methylation.

Furthermore, while the methylation levels of e‐iNOS increased in this protocol, p‐iNOS remained unaltered after exercise. Because of different exercise protocols and tissue sampling, it is currently difficult to establish a consensus on the effects of exercise on DNA methylation changes. Finally, the addition of different hypoxic or ischemic stimuli did not impact these responses. Previously, it was found that incremental exposure to hypoxia induces alterations in the epigenome, promoting acclimatization to altitude (Childebayeva, Harman, et al., 2019). In this study, the authors found lower global methylation levels (LINE‐1) at high altitudes compared to baseline, as well as differential methylation of several markers (e.g., PPARa, EPAS1, EPO, and RXRa) depending on the altitude level and the duration of the exposition (i.e., time window of several days compared to our study). The authors also found a correlation between EPO methylation levels and systolic blood pressure, as well as between hemoglobin content and the methylation profile of RXRa (Childebayeva, Harman, et al., 2019). The absence of effects of hypoxia on DNA methylation in our study should be explained by the latency that would be necessary for the exhibition of adaptations related to hypoxia, as well as the short exposure. Indeed, the study from Childebayeva suggests that changes in the epigenome could be linked to altitude acclimatization. However, short intermittent exposures to hypoxia do not seem to promote acclimatization, as the hypoxic ventilatory response and oxygen saturation levels remain unchanged after 7‐hourly exposures on consecutive days (Treml et al., 2020). In combination with exercise, altitude acclimatization could be induced (Benoit et al., 1992), but several sessions were employed. Thus, it is probable that the current protocol did not elicit altitude acclimatization processes and cannot be compared to the study by Childebayeva and colleagues. In perspective, studies must evaluate DNA methylation responses at different time points and if the modifications in DNA methylation impact gene expression. Furthermore, more exhaustive techniques such as DNA methylation microarrays should shed light on exercise‐induced changes in DNA methylation with a wide range of CpG sites analyzed.

This study faces a limitation concerning its ability to detect differences in DNA methylation modifications between conditions. Despite the potential regulatory impact of exercise‐induced methylation changes at individual CpG sites (Bajpeyi et al., 2017; Barrès et al., 2012; Hunter et al., 2019), identifying relevant methylation patterns within short sequences of a single gene remains challenging. Furthermore, the method utilized lacks single‐CpG resolution when multiple CpG sites are found in the amplified sequence. As such, in this study, each sequence contains at least two CpG sites potentially cut by restriction enzymes, preventing the determination of which CpG site is responsible for the observed effects. Additionally, the possibility of synergy or antagonism between both CpG dinucleotides adds complexity to the interpretation. For instance, our experimental approach would not detect cases where the first site undergoes hypermethylation while the second site experiences hypomethylation, or vice versa. This scenario could possibly explain the lack of observable differences in p‐iNOS. While saliva exhibits lower cellular heterogeneity compared to blood, the characterization of salivary cell composition would have been an important parameter to control to guarantee sample uniformity. The proportion of each cell type could have influenced the results. It is also worth noting that bacterial DNA contamination cannot be excluded. Furthermore, despite the evident advantages of using saliva for DNA methylation studies, such as its noninvasive collection process and high yield and quality of extracted DNA, this biofluid is not directly involved in physical exercise. Hence, it merely serves as a surrogate tissue. Due to the tissue‐specific nature of DNA methylation changes, the current results cannot be extrapolated to other tissues such as skeletal muscle. In perspective, it would be worthwhile to investigate the effects of multiple SIT sessions conducted under these conditions over several weeks on epigenetic markers.

## 5 CONCLUSION

In conclusion, this study provides evidence that all‐out exercise intervals induce increased global DNA methylation in human saliva. Furthermore, the introduction of either systemic or local hypoxic stresses did not yield discernible differences in global DNA methylation. However, the increase in global DNA methylation tended to be more prominent in BFR compared to the other conditions. One of the limitations of this study is the fact that only short sequences within the promoter and enhancer of a single gene were evaluated. Hence, the results show an incomplete picture of the targeted genomic region. Furthermore, the technique that was employed (methylation‐sensitive restriction enzymes) lacks the precision to identify methylation changes at individual CpG sites within the sequences of interest. As a result, alterations in DNA methylation pattern remained indiscernible. Further investigation should explore how other tissues, such as skeletal muscle, respond to systemic or local hypoxic acute or repeated exposures. In perspective, studies should also evaluate if the modifications in DNA methylation impact gene expression.

## FUNDING INFORMATION

This research received no specific grant from any funding agency in the public, commercial, or not‐for‐profit sectors. Open access funding was provided by the University of Lausanne.

## CONFLICT OF INTEREST STATEMENT

No conflict of interest, financial or otherwise, is declared by the authors.

## DISCLOSURE STATEMENT

The authors did not use any generative artificial intelligence tools in the preparation of their article.

## ETHICS STATEMENT

The participants gave written consent to participate in the study, which was validated by the cantonal research ethics committee (“Commission cantonale d'éthique de la recherche sur l'être humain”, canton de Vaud, CER‐VD 2021‐00597), and was performed in accordance with the ethical standards of the 1964 Declaration of Helsinki.

## Data Availability

The data that support the findings of this study are available on request from the corresponding authors, FB, HB and AMJS.
